# Nectarivorous Bird Emphysematous Ingluvitis (NBEI): A Novel Disease in Loriinae Birds Associated With *Clostridium perfringens* Infection

**DOI:** 10.3389/fvets.2020.606112

**Published:** 2020-11-05

**Authors:** Andrew F. Rich, Flavia Zendri, Taiana Costa, Dorina Timofte, Gabby J. Drake, Hannah Rowland, Ian Ashpole, Andrew Moore, Julian Chantrey

**Affiliations:** ^1^Department of Veterinary Anatomy, Physiology and Pathology, Institute of Infection, Veterinary and Ecological Sciences, University of Liverpool, Leahurst Campus, Neston, United Kingdom; ^2^North of England Zoological Society (Chester Zoo), Chester, United Kingdom; ^3^Oakhill Veterinary Centre, Goosnargh, United Kingdom

**Keywords:** *Clostridium perfringens*, emphysematous ingluvitis, nectarivorous, lory, lorikeet

## Abstract

A retrospective study revealed ten cases of emphysematous ingluvitis in Loriinae birds from two zoological collections between 2009 and 2020. Common clinical features were sudden death with gas distention of the crop, subcutaneous cervical emphysema and poor body condition, but also included collapse, hypothermia and abandonment. Macroscopic examination revealed moderate crop enlargement, distention and thickening with minimal intraluminal content, and moderate to severe submucosal to transmural gas-filled cysts (emphysema). Histopathology identified widespread transmural multifocal to coalescing empty pseudo-cystic cavities with lytic necrosis, pyo-/granulomatous inflammatory infiltrates, epithelial ulceration, parakeratotic hyperkeratosis, epithelial ballooning degeneration, and occasional intralesional rod-shaped bacteria. The lesion may have impaired the birds' ability to ingest food, resulting in suboptimal body condition. Necrotizing to granulomatous aspiration pneumonia was also a feature in some cases. Anaerobic bacterial culture of four crops identified *Clostridium perfringens* with associated toxin genes for alpha and occasionally beta2 toxin (*cpa* and *cpb2* genes respectively), by PCR analysis of bacterial isolates cultured from fresh or frozen tissue. *C. perfringens* was identified as the common etiological agent of emphysematous ingluvitis in crop and/or liver (six out of ten birds), and type A was confirmed in five birds. *C. perfringens* was not detected in the crop nor liver of two unaffected Loriinae birds. This is the first publication that characterizes nectarivorous bird emphysematous ingluvitis (NBEI), attributes *C. perfringens* as an etiological agent, and highlights this novel disease as an important cause of death in Loriinae birds, particularly in nestling and fledgling stage of development, but also in older lorikeets and lories.

## Introduction

Lorikeets and lories are nectarivorous birds (subfamily *Loriinae*, family *Psittacidae*) indigenous to Australasia, that are commonly kept in zoological collections due to their active nature and brightly colored, compact profile (1). In captivity, diets are typically composed of nectar substitutes, concentrates/pellets, fruits, flowers and occasional small insects (1, 2). Many are involved in captive breeding programs (3), and effective disease surveillance is therefore important for establishing resilient captive populations.

There are increasing reports of clostridial diseases in wild or captive wild birds, with Tyzzer's disease (*Clostridium piliforme*) (4, 5), ulcerative enteritis/quail disease (*C. colinum*) (6), *C. tertium*–associated enteritis plus *C. perfringens*-associated enteritis (7, 8) and necrotic enterocolitis (9) being identified in lorikeet or lory species. Two sources have recognized a novel disease within rainbow lorikeets (*Trichoglossus haematodus haematodus*) (10) and red-flanked lorikeets (*Chamorsyna placentis*) (11), characterized macroscopically by thickened crops with nodular-to-diffuse mucosal nodules and gas-filled bubbles, and histologically as emphysematous ingluvitis. However, an etiological agent has not been identified in these cases. Another source describes crop lymphangiectasia in a hybrid rainbow lorikeet, which externally showed a thickened crop wall, and endoscopically the crop, and distal cervical esophageal mucosa was covered by numerous 2–3 mm diameter clear cavities (12). Histologically, abundant variably sized empty pseudo-cystic spaces severely expanded the submucosa and were occasionally surrounded by multinucleated giant cells (12). This reported lesion was grossly and histologically identical to emphysematous ingluvitis. These lesions have been histologically compared to pneumatosis cystoides intestinalis and gastric pneumatosis (emphysematous gastritis) in humans and other animals (13, 14).

Emphysematous diseases are commonly associated with *Clostridium* spp. infection (15), most notably malignant edema in various species (15) and blackleg in cattle (16), with other sporadic reports including emphysematous gastritis in horses (17) and cats (18), and *C. perfringens*-associated cystitis in a dog (19).

This retrospective analysis aims to characterize the clinical, pathological and bacteriological features of emphysematous ingluvitis in ten Loriinae birds.

## Materials and Methods

### Retrospective Study

This study was performed using the University of Liverpool Veterinary Pathology Diagnostic Service database for case submissions of Loriinae birds between 2009 and 2020. All cases containing the term “emphysematous ingluvitis” as a histomorphological diagnosis were included. Information regarding the case history, clinical presentation, animal husbandry/environmental conditions, plus bacterial culture and molecular typing of each case was retrieved and analyzed.

All cases were submitted for routine post-mortem examination (PME) within 24–48 h of death, delivered cold (<4°C). Tissue samples were collected during PME, fixed overnight in 10% neutral buffered formalin and submitted for routine histopathological analysis. They were trimmed, paraffin-embedded, sliced into 3 μm histologic sections for fixation on glass slides, and stained with hematoxylin (Hematoxylin; TCS Biosciences Ltd.) and eosin (Eosin; TCS Biosciences Ltd.), Gram, and, occasionally, with Periodic acid-Schiff (PAS). Histopathological analysis was conducted using light microscopy.

### Bacterial Culture and Molecular Typing of *C. perfringens* Isolates

Direct aerobic and anaerobic bacterial culture of fresh crop (*n* = 2) and liver (*n* = 3) tissue was achieved on 5% sheep blood agar and fastidious anaerobe agar (all media Oxoid™, Basingstoke, UK), incubated at 37°C for 48 h. Enrichment in cooked meat medium was also performed on frozen crop (*n* = 2) and liver (*n* = 3) tissues, allowing pre-reduction of the broth for 1 h under anaerobic conditions before inoculation onto neomycin blood agar after 48 h.

Toxinotyping of *C. perfringens* bacterial cell lysates obtained by boil preparation from cultured fresh and frozen tissues was performed in triplicate by multiplex PCR assay for the toxin genes *cpa, cpb, etx, iA, cpe*, and *cpb2* (20). The *netB* toxin gene was investigated according to Keyburn et al. (21). All amplified products were resolved by electrophoresis through a 1.5% (w/v) TAE agarose gel.

### Molecular Typing of *C. perfringens* in Formalin-Fixed and Paraffin Wax-Embedded Tissues

Formalin fixed and paraffin wax-embedded (FFPE) crop (*n* = 9) and liver (*n* = 10) tissues in cases positive for NBEI were investigated for *C. perfringens* by PCR assays. Crop and liver FFPE tissues from two additional birds not showing post-mortem evidence of emphysematous ingluvitis were included as negative controls. FFPE sections (2 × 5 mm) cut using a punch biopsy needle underwent total DNA extraction using QIAamp® DNA Mini kit (Qiagen, Hilden, Germany) according to the manufacturer's protocols. *GAPDH* was selected as a reference gene to assess quality of avian DNA extracted from FFPE tissues as per Le Maitre et al. (22). FFPE samples were subsequently screened for *C. perfringens* using specific 16S rRNA gene primers according to Wu et al. (23).

## Results

### Retrospective Study

Ten Loriinae birds from four species were included in this study (B1–B10, Table 1). They included seven Mindanao lorikeets (*Trichoglossus johnstoniae; six* nestlings, one fledgling), a rainbow lorikeet (*Trichoglossus moluccanus;* fledgling**)**, a yellow-backed chattering lory (*Lorius garrulous flavopalliatus*; juvenile) and a purple-naped lory (*Lorius domicella;* adult). The cases were submitted from two zoological collections (Z1 and Z2) 2009 and 2020 at various times of the year. *Trichoglossus* specimens exhibited the lesion in the nestling and fledgling stages (post-6 weeks), with a mean age of 39 days (5.5 weeks), whilst the Lorius genera exhibited the lesion later in development or adulthood. Details on avian husbandry and environmental conditions are presented in Supplementary Table 1.

**Table 1:** Signalment, the presence of macroscopic and histopathological lesions of birds presented with emphysematous ingluvitis and positive/negative detection of *C. perfringens*.

**Cases**	**Species**	**Age**	**Zoological collection**	NBEI macroscopic lesions^*^	NBEI Histological lesions^**^	**Evidence of aspiration bronchopneumonia**	***C. perfringens* isolation**.
Control 1	ML	1M 28D	Z1	-	-	-	-
Control 2	ML	7Y 7M 7D	Z1	-	-	-	-
B1	YBCL	6M 24D	Z1	+	+	-	N.A.
B2	ML	21D	Z1	+	+	-	N.A.
B3	ML	23D	Z1	+	+	+	+
B4	ML	1M 28D	Z1	+	+	-	+
B5	PNL	10Y 11M 5D	Z1	+	+	+	N.A.
B6	RL	1M 12D	Z2	+	N.A.	+	+
B7	ML	1M 7D	Z1	+	+	+	N.A.
B8	ML	1M 3D	Z1	+	+	-	+
B9	ML	1M 4D	Z1	+	+	-	+
B10	ML	2M 1D	Z1	+	+	+	+

*ML, Mindanao lorikeet; RL, Rainbow lorikeet; YBCL, Yellow-backed chattering lory; PNL, Purple naped lory; N.A., not available; Y, years; M, months; D, days*.

* *Macroscopic lesion in crop compatible with NBEI: moderate to severe distension, moderately thickened wall, minimal intraluminal content, and moderate to severe submucosal to transmural gas-filled cavities (emphysema)*.

** *Histological lesions in crop compatible with NBEI: (1) subepithelial pseudo-cyst formation, (2) lytic necrosis, (3) granulomatous to pyogranulomatous ingluvitis, with (4) intra-lesional bacteria*.

Five Mindanao lorikeets (B2, B4, B8, B9, and B10) and the yellow-backed chattering lory (B1) presented with sudden death. No clinical signs were identified prior to death, but four of the five lorikeets were parent-reared nestlings and therefore, regular visual assessment was not feasible.

On two separate occasions (B3 and B7), nestling Mindanao lorikeets presented underweight, collapsed, hypothermic and abandoned in the nest. The main clinical signs identified were gas distention of the crop and cervical subcutaneous emphysema. Birds were hospitalized in an intensive care incubator (Model TLC-5M, Brinsea) where crop aspiration and subcutaneous fluid therapy were performed. B7 received parenteral metoclopramide (Emeprid® 5 mg/ml, Ceva Animal Health Ltd., Bucks, UK). Both cases were unresponsive to treatment and were euthanized within 12 h of presentation.

The rainbow lorikeet (B6) was one of several parent-reared chicks that initially presented as bright and active, but with crop distention. This progressed to lethargy and weakness or death within 24–48 h. Only one chick was submitted for necropsy from a series of similar clinically affected cases at Z2 due to the enclosure's high temperature (24°C) resulting in rapid autolysis of the other chicks.

The purple-naped lory (B5) exhibited lethargy, anorexia, dehydration and emaciation. It was treated with parenteral marbofloxacin (Marbocyl® 2%, Vetoquinol UK Ltd., Towcester, UK), subcutaneous fluid therapy including glucose and crop tubing with critical care formula (CCF Critical Care Formula; Vetark, Winchester, UK). Crop distention and palpable crop thickening developed following 5 days of treatment. Despite initial improvement, the bird deteriorated by day 4 and was euthanized on day 6.

### Macroscopic Findings

**Figure 1:**
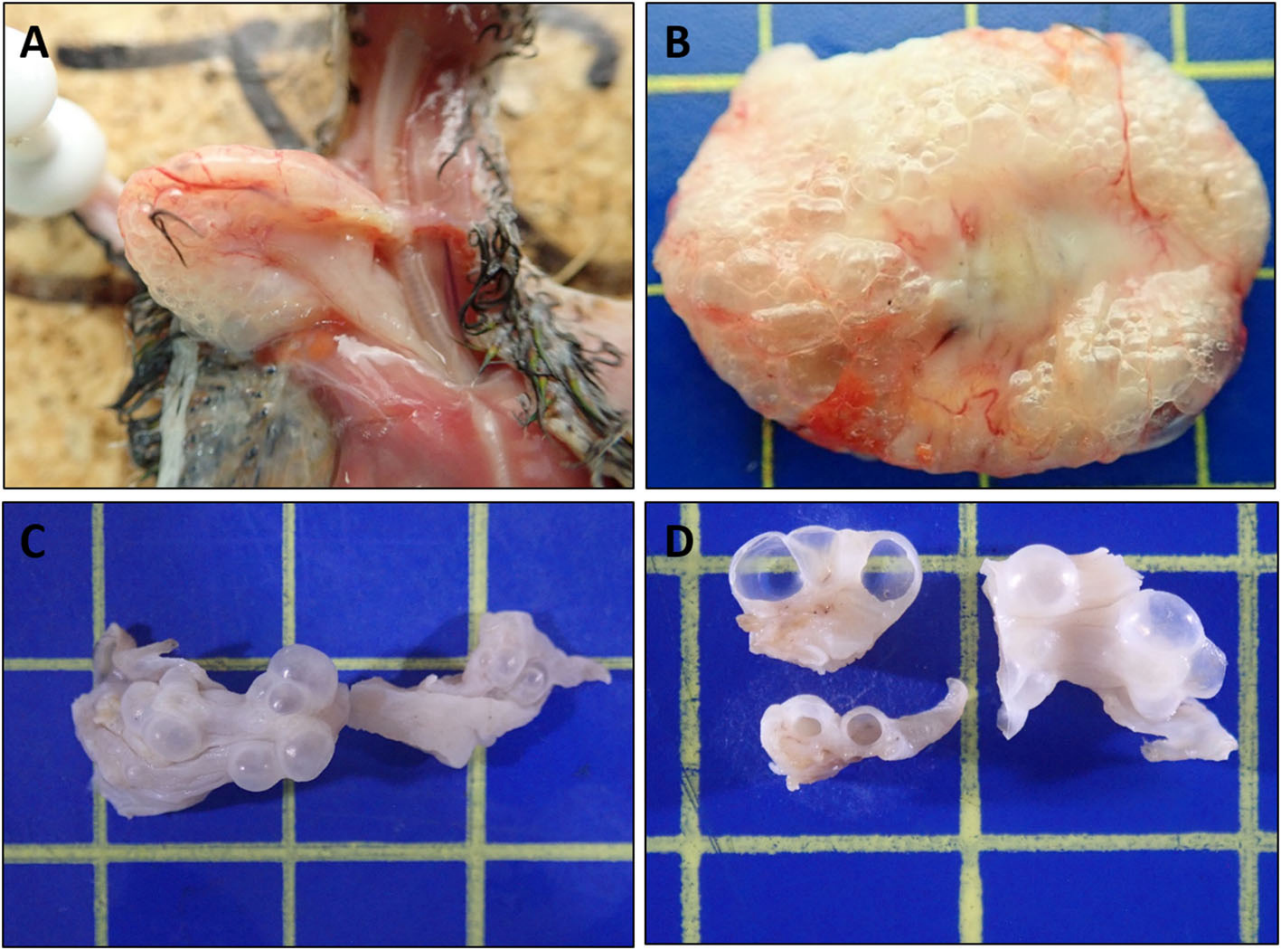
Macroscopic features of Nectarivorous Bird Emphysematous Ingluvitis (NBEI) in Loriinae Birds **(A–D)**. **(A)** Case 8, emphysematous crop [*in-situ*] and **(B)** Case 9, emphysematous crop [fresh excised tissue]; both exhibiting diffuse submucosal to transmural gas-filled cavities (emphysema), distension and mural thickening. **(C,D)** Case 10, emphysematous crop [10% formalin fixed excised tissue]. **(C)** Intact multifocal to coalescing exophytic gas-filled cavities and **(D)** cut surfaces of cavities. (Blue cutting board used as background: each box 1cm^2^).

All ten birds exhibited similar macroscopic lesions of the crop (Table 1), characterized by moderate to severe distension, moderately thickened walls, minimal intraluminal contents and moderate to severe submucosal to transmural gas-filled cavities (emphysema) (Figures 1A–D). The crops were buoyant when placed in 10% formalin solution (Supplementary Figure 1). There was occasional diffuse moderate crop erythema/congestion (B1, B5, B8, and B9) and red to yellow adherent mucosal diptheric pseudomembranes (B2 and B5). Apart from B6, no other lesions were identified macroscopically nor could be attributed to the subjects' deaths. B6 exhibited concurrent multifocal variably size up to 3 mm in diameter, circumferential, white to tan firm nodules (necrogranulomas) within the pulmonary parenchyma, and a focal, round (~5 mm in diameter), white to tan firm hepatic nodule (bacterial granuloma).

### Histopathological Examination

**Figure 2:**
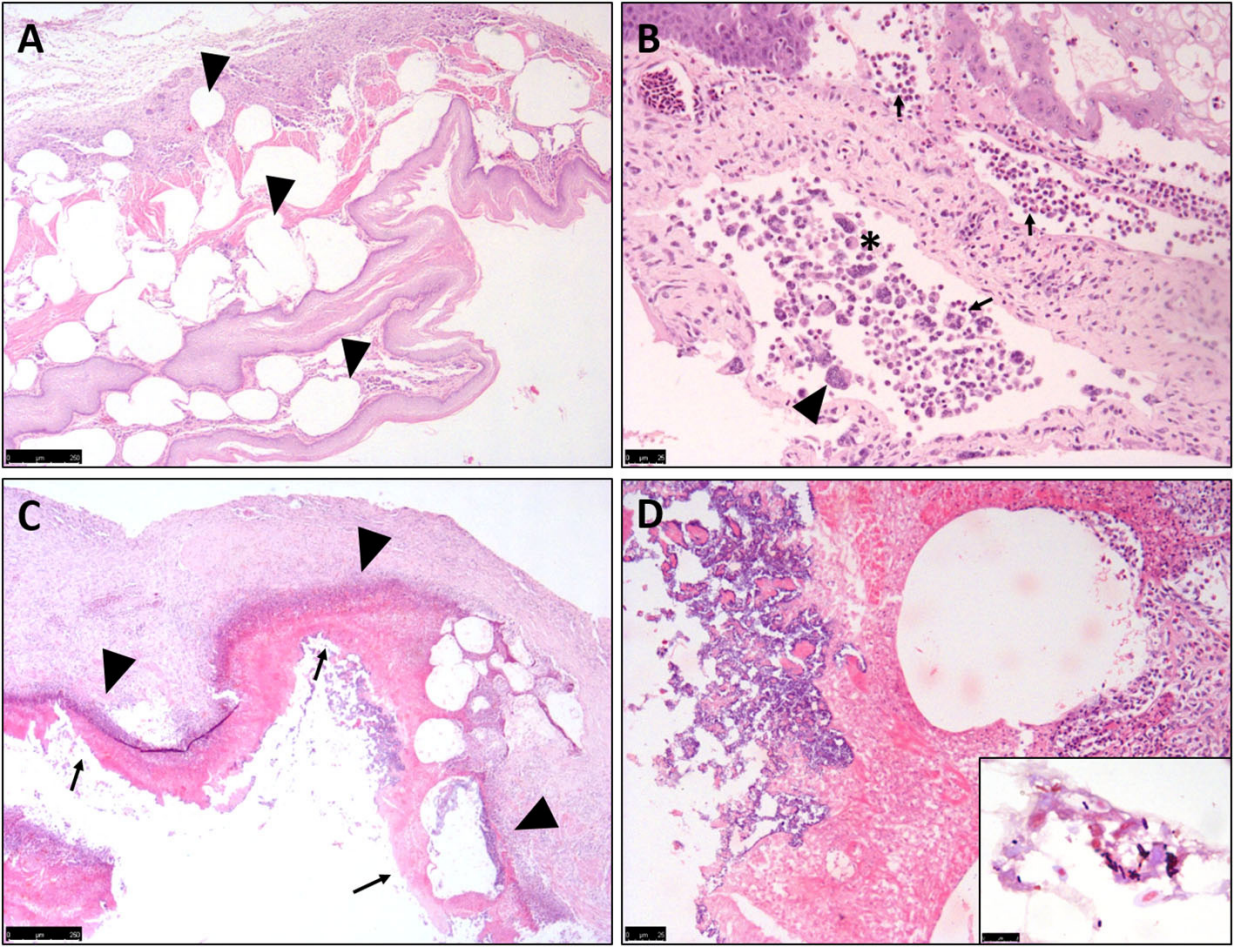
Histopathological features of Nectarivorous Bird Emphysematous Ingluvitis (NBEI) in Loriinae Birds. **(A–D)** Representative photomicrographs of formalin-fixed section of crop. **(A)** Numerous sub-epithelial pseudo-cystic cavities (emphysema) [arrowheads] within the lamina propria, submucosa, muscularis mucosae and sub-adventitial layer (Case 9, H&E stain, Obj. 2×). **(B)** Multifocal to coalescing, moderate, mixed inflammatory infiltrations of mostly macrophages [asterix], heterophils [arrows] and occasional multinucleated giant cells (Langhans-type) [arrowheads] (Case 2, H&E stain, Obj. 20×). **(C)** Multifocal epithelial degeneration, sloughing and occasional mucosal ulceration superimposing inflammation [arrowheads] resulting in pseudomembrane formation [arrows] (Case 8, H&E stain, Obj. 4×) and **(D)** Multifocal mixed bacteria (Case 8, H&E stain, Obj. 40×) [Inset: Higher magnification of Gram-positive, slightly curved bacillary, *Clostridium* spp.-like bacteria (Case 8, Gram stain, Obj. 100×)].

All ten birds exhibited similar histopathological lesions in the crop (Table 1), although distribution and severity varied. The major lesion in the crop was a diffuse transmural emphysematous process characterized by small (40 μm diameter) to large (2–3 mm) multifocal to coalescing empty pseudo-cysts with no epithelial lining, within the lamina propria, submucosa, muscularis mucosae and sub-adventitial layer (Figure 2A). This supports the macroscopic changes of submucosal gas-filled cavities. Concurrent multifocal to coalescing, moderate, mixed inflammatory infiltrations of mostly macrophages, heterophils and occasional multinucleated giant cells (Langhans-type) (Figure 2B) were observed in all cases within the lamina propria, admixed with necrotic cellular debris, moderate extracellular edema and marked fibrosis. Multifocal regions of epithelium showed large foci of intracellular edema (ballooning degeneration), sloughing and occasional mucosal ulceration, and parakeratotic hyperkeratosis superimposed on the inflammation (Figure 2C). The crop lumina contained mild to severe accumulations of necrotic cellular debris (lytic necrosis) degenerate heterophils and macrophages, sloughed epithelium and occasional small to large abundant numbers of mostly Gram-positive rod-shaped bacteria (suggestive of clostridia-like organisms) (Figure 2D), with some cases exhibiting mixed Gram-positive and Gram-negative coccobacillary bacteria which are incorporated into pseudomembranes.

The second most common finding was necrogranulomatous pneumonia, observed in five cases, occasionally associated with aspirated food material (Table 1).

B6 had a moderate multifocal necrogranulomatous pneumonia and a severe, multifocal to coalescing necrotizing hepatitis, associated with *Salmonella* spp. infection, which was co-isolated from the liver along with *C. perfringens* type-A.

*Trichomonas* spp. and protozoal organisms were not detected histologically in any case.

### Bacterial Culture

Aerobic and anaerobic bacterial culture was performed on fresh crop (*n* = 2) and fresh liver (*n* = *3*), frozen crop (*n* = *2*) and frozen liver (*n* = *3*) tissue samples (Table 2). For three cases, only aerobic cultures of fresh crop were performed (historical records).

**Table 2:** Bacterial culture and *C. perfringens* molecular toxinotyping results from birds with NBEI.

**Cases**	**Species**	**Bacterial culture**		**Molecular toxinotyping**		
		***C. perfringens* (organ, storage)**	**Other bacterial isolates (organ, storage)**	***cpa* gene (α-toxin, type A)**	***cpb2* genes (β2-toxin, type A^**β2**^)**	***NetB* gene (NetB toxin)**
Control 1	ML	– (Liver, fresh) Crop: N.A.	*Enterobacter asburiae* and *Staphylococcus equorum* (Liver, fresh)	–	–	–
Control 2	ML	– (Crop/Liver, fresh)	*E. cloacae, E. coli* and *Kocuria* spp. (Crop, fresh); *S. gallinarum, E. coli* and *E. faecalis* (Liver, fresh).	–	–	–
B1	YBCL	N.A.	*K. pneumoniae pneumoniae* and *Enterococcus* spp. (Crop, fresh), Liver: N.A.	N.A.	N.A.	N.A.
B2	ML	Crop/Liver: N.A.	Crop/Liver: N.A.	N.A.	N.A.	N.A.
B3	ML	+ (Crop, frozen), – (Liver/frozen).	-	+	–	–
B4	ML	+ (Crop/Liver, frozen)	-	+	+ (crop/liver)	–
B5	PNL	N.A.	Non-haemolytic *Staphylococcus* spp. and *E. faecalis* (Crop/fresh), Liver: N.A.	N.A	N.A	N.A
B6	RL	+ (liver, frozen). Crop: N.A.	*C. septicum* (crop, frozen), *Salmonella* spp. (Liver, fresh). Crop: N.A.	+	+	–
B7	ML	N.A.	*E. cloacae, Candida* spp. and *Leuconostoc* spp. (Crop, fresh), Liver: N.A.	N.A	N.A	N.A
B8	ML	+ (Liver, fresh). Crop: N.A.	Non-haemolytic *E.coli* (liver, fresh). Crop: N.A.	+	–	–
B9	ML	+ (Crop/Liver, fresh)	Non-haemolytic *E.coli* (crop/liver, fresh).	N.A	N.A	N.A
B10	ML	+ (Crop, fresh), – (Liver, fresh)	*E. cloacae* and *S. sciuri* (Crop, fresh), *Klebsiella oxytoca, S. equorum, S. sciuri* and *E. faecalis* (Liver, fresh).	+	–	–

*ML, Mindanao lorikeet; RL, Rainbow lorikeet; YBCL, Yellow-backed chattering lory; PNL, Purple naped lory; +, positive; –, negative; N.A., not available; E. coli, Escherichia coli; S., Staphylococcus*.

*C. perfringens* was isolated from six birds: two fresh (B9 and B10) and two frozen (B3 and B4) crops, plus two fresh (B8 and B9) and two frozen (B4 and B6) liver tissues (Table 2). Heavy growths of *C. perfringens* were cultured from the fresh and/or frozen crop tissues (B3, B4, B9, and B10). Cultured fresh and/or frozen liver tissues from three of the birds yielded light mixed aerobic and/or anaerobic bacterial growths including *Escherichia coli* (B8 and B9), *Salmonella* spp. (B6), and *C. septicum* (B6) isolated alongside *C. perfringens* (Table 2). *C. perfringens* was isolated from all birds for which anaerobic culture was performed. Anaerobic culture of crop and liver was not performed for cases B1, B5, and B7, and no fresh or frozen tissue was available for case B2.

Other opportunistic pathogens isolated from the affected crops included *Candida albicans, Enterobacter cloacae*, and *Leuconostoc* spp., plus non-haemolytic *E. coli* with *C. perfringens* (B9); *Klebsiella pneumoniae* subspecies *pneumoniae* and *Enterococcus* spp. (B1) and non-haemolytic *Staphylococcus aureus* and *Enterococcus faecalis* (B5).

### Molecular Typing of *C. perfringens* Isolates

Six of the eight *C. perfringens* isolates (crop = 3; liver = 3) from five birds were available for molecular typing. Besides alpha toxin (CPA) present in all *Clostridium* isolates, B4 (crop and liver) and B6 (liver) carried CPB2 encoding the β2-toxin (type A^β2^) (Table 2). All bacterial isolates tested negative for the *NetB* toxin gene.

### Molecular Typing of *C. perfringens* in Formalin-Fixed and Paraffin Wax-Embedded Tissues

PCR analysis of DNA extracted from the FFPE tissues failed to identify either *GAPDH* housekeeping gene products or *C. perfringens* 16S rRNA, suggesting that the DNA extraction was inadequate.

## Discussion

All NBEI cases exhibited four common histopathological features: (1) subepithelial pseudo-cyst formation, (2) lytic necrosis, (3) granulomatous to pyogranulomatous ingluvitis, with (4) multifocal intralesional bacteria. Considering their consistency, these features can be considered typical for diagnosis of NBEI histologically, and cannot be mistaken for other diseases in nectarivorous birds. The poor body condition and aspiration pneumonia most likely reflect dysphagia and poor gastrointestinal motility caused by NBEI.

Pneumatosis cystoides intestinalis (PCI) and gastric pneumatosis (GP) in humans and other animals (13, 14) have been identified as relatable conditions, and share comparable features, specifically, multiple gas-filled cystic spaces frequently located within the gastrointestinal serosal or mucosal layers, and both lesions exhibit macroscopic and histological “gas-bubble” features. Differences mainly include organ distribution where PCI affects stomach, intestines, mesentery, omentum, and adjacent lymph nodes, whilst GP is localized to the stomach wall (a subset of PCI). Idiopathic eosinophilic gastritis has been also associated with GP in lemurs (14). On this basis, NBEI could possibly be included as a novel subset of PCI. Primary PCI is considered idiopathic, however secondary forms may be induced by various non-infectious and infectious factors (13, 14), including anaerobic bacteria, e.g., *Clostridium* spp. due to their ability to colonize gastrointestinal walls and metabolically produce hydrogen gas (13, 14, 24), similarly to NBEI.

*C. perfringens* is a Gram-positive anaerobic rod-shaped bacterium that is categorized into seven types (A-G), according to the production of major toxins, specifically alpha (CPA), beta (CPB), epsilon (ETX) and iota (ITX), plus enterotoxin (CPE) and NetB toxin (NetB) (25). Anaerobic culture was only performed in six out of the ten specimens, all of which were positive for *C. perfringens*, with A and A^β2^-subtypes found in three and two of these five cases respectively, highly suggesting an association between *C. perfringens* type A infection and the development of NBEI (26). *C. perfringens* was isolated in heavy growths from Case B9; however, the samples were misplaced before toxinotyping could be performed. Direct PCR of FFPE tissues failed, therefore the presence of the bacterium could not be detected in the remaining four birds for which anaerobic culture could not be performed.

In cases B4 and B9, identical isolates were cultured from both crops and livers, suggesting that in severe cases, systemic circulation of the bacteria may occur *via* the enteric-portal system resulting in death before hepatic lesions develop. Examination of negative control crop and liver tissues by bacterial culture revealed that *C. perfringens* was not present in the absence of NBEI in these two control birds. Both control and NBEI birds showed similar states of preservation, therefore *C. perfringens* type A potentially may not be a commensal bacterium found in the crop or liver of nectarivorous birds, however, without additional crop flora samples of non-NBEI lorikeets, this cannot be definitively confirmed. In B6 and B8, although frozen crops were not available, the livers of both birds were positive for *C. perfringens* types A^β2^ and A, respectively. Whilst not directly attributable to the NBEI, the presence of *C. perfringens* in these cases corroborates with the association between *C. perfringens* type A and NBEI.

Alternatively, as hepatic lesions were absent, *C. perfringens* may have been found in the liver due to bacterial translocation occurring post-mortem from the gastrointestinal tract where it could have been residing ante-mortem as an opportunistic pathogen or commensal, as occurs in chickens (27). However, *C. perfringens* has been associated with necrotic enterocolitis in lorikeets (9, 28), which was not observed in our cases, therefore hepatic *C. perfringens* may suggest systemic circulation from the crop to the liver in cases of NBEI, in the absence of necrotic enterocolitis. Our negative controls would support this theory rather than autolytic invasion.

Other crop microbiological isolates from some of our cases included *C. albicans, E. coli, K. pneumoniae* subspecies *pneumoniae, Enterobacter* spp., *Enterococcus* spp. and *S. aureus*, which are all implicated in emphysematous gastritis in humans (29–33). These bacteria can be gas producers, and consequently whilst *C. perfringens* is the most probable significant agent in our case series, these other pathogens may still be capable of inducing similar emphysematous ingluvitis. However, *E. cloacae* and *E. coli* were detected within the normal crop of a negative control bird and so these may represent opportunist pathogens or non-pathogenic commensals.

The limitation of the study was the unavailability of frozen crop and/or liver tissues for anaerobic culture in some cases (including B6, B8 [crop], B1, B2, B5, and B7 [crop and liver]). Although PCR analysis of FFPE crop and liver tissues was attempted, this proved unrewarding as all cases produced negative or negligible results. Therefore, *C. perfringens* cannot be discounted as an etiological agent for NBEI in cases B1, B2, B5, and B7, as clostridia-like bacteria were detected histologically.

Our findings suggest that emphysematous ingluvitis appears only to affect nectarivorous species of the *Trichoglossus* or *Lorius* genera and has not been detected in other genera within the *Psittaculidae* family previously. *Trichoglossus* specimens appear to exhibit the lesion in the nestling and fledgling stages (post-6 weeks), with a mean age of 39 days (5.5 weeks), whilst the *Lorius* genera exhibit the lesion later in development or adulthood. This interpretation, however, may be biased by case submission, different breeding activity in the different species and limited previous surveillance. The rainbow hybrid lorikeet recorded by Perpiñán et al. (12) with similar findings was an 80-day-old fledgling, and our two lories showed the lesion around 6.5 months to 11 years old; consequently, an expected wider age range for NBEI may be appropriate. Importantly, all cases exhibit poor body condition suggesting that the crop lesion readily impaired the birds' ability to eat, or alternatively, poor body condition or a nutritional issue could pre-dispose birds to NBEI. In very young vulnerable individuals, reliant on parents to provide nutrition and with limited body reserves, this inability to ingest would result in faster disease progression and higher mortality; therefore, the nestling/fledgling period may represent the period with highest risk of mortality. This coincides with a developmental diet change period in which nestlings/fledglings convert from a high protein (insectivorous) diet to a high sugar diet (34), which may be a potential disease risk factor in some zoos. Such a sudden change in protein availability may provoke *C. perfringens* to seek other protein-sources, namely the animal host cells, as the bacterium has been demonstrated to be “flesh-eating” by obtaining various host substrates by producing an array of degradative enzymes and toxins inducing massive tissue necrosis (35).

Nectar spoilage induced by the warmer weather of the breeding season was a proposed risk factor for the parent-reared Mindanao lorikeet nestlings and fledglings in Z1; however, bacterial culture of the nectar provided to the lorikeets in 2019 did not yield any *C. perfringens*. This may suggest that poor quality or foul nectar may pre-dispose overgrowth of *C. perfringens*, as occurs in rabbits with epizootic rabbit enteropathy in which dysbiosis (imbalance of natural gut flora) and *Clostridium* spp. overgrowth have been proposed as associated risk factors (36).

Proposed investigations into this condition include fulfillment of Koch's postulates, disease pathogenesis and evaluation of disease risk factors to enhance diagnosis, prevention, and control of this disease in zoological collections.

## Data Availability Statement

The original contributions presented in the study are included in the article/Supplementary Material, further inquiries can be directed to the Corresponding author.

## Ethics Statement

The animal study was reviewed and approved by University of Liverpool's Research Ethics Committee (VREC946–Lorikeets).

## Author Contributions

AR, FZ, TC, DT, and JC designed the study. AR, JC, GD, HR, and IA identified the archived specimens. AR, TC, and JC examined and photographed the macroscopic specimens. AR and JC examined and photographed the histopathological sections. FZ and DT performed the bacteriology experiments. GD, HR, IA, and AM provided the clinical and husbandry data from Chester and Blackpool Zoo, and edited these sections. AR, FZ, GD, and HR prepared the manuscript. AR, FZ, DT, GD, HR, TC, and JC analyzed the data. All revised the manuscript.

## Conflict of Interest

The authors declare that the research was conducted in the absence of any commercial or financial relationships that could be construed as a potential conflict of interest.
