# Supplementary material for: Nectarivorous Bird Emphysematous Ingluvitis (NBEI): A Novel Disease in Loriinae Birds Associated With Clostridium perfringens Infection
Source: Front Vet Sci. 2020 Nov 5;7:606112. doi: 10.3389/fvets.2020.606112 (PMC7674492; doi:10.3389/fvets.2020.606112)
Supplement: Supplementary file 1 [file Data_Sheet_1.docx]

Supplementary Material

**Supplementary Table 1**: Animal husbandry and environmental conditions of the two zoological collections where cases of emphysematous ingluvitis originated.

| **Husbandry / environmental conditions** | **Zoological collection** | |
| --- | --- | --- |
|  | **Z1** | **Z2** |
| **Cases** | B1-B5, B7-10 and both control birds. | B6 |
| **Enclosure** | Indoors (24^ᵒ^C) and outdoors access. Serviced twice daily. | Indoor access (24^ᵒ^C). Serviced twice daily. |
| **Husbandry** | Purple naped lories and Mindanao lorikeets were kept as individual breeding pairs; breeding between aviaries was not synchronized. Yellow-backed chattering lories were kept communally and as pairs. | Group of 34 rainbow lorikeets  Access to 8 nest boxes; individual breeding pairs occupied all boxes at any one time. |
| **Diet** | Commercially available nectar mix; replenished once daily outside of the nesting season and twice daily during the nesting season. Fresh fruit (apples, pears, grapes, papaya, strawberries and banana); offered once or twice daily. | Commercially available nectar mix and fresh fruit (apples, pears, grapes, papaya, pomegranate and banana); offered twice daily. |

**Supplementary Figure 1.** Case 10, emphysematous crop freely floating in 10% formalin solution due to sub-epithelial air-filled pseudocysts.

**
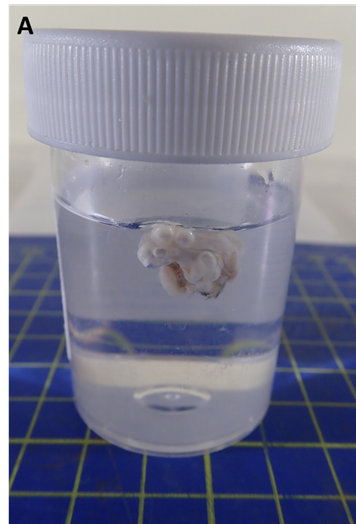
**
